# Bone allograft impregnated with tobramycin and vancomycin delivers antibiotics in high concentrations for prophylaxis against bacteria commonly associated with prosthetic joint infections

**DOI:** 10.1128/spectrum.00414-24

**Published:** 2024-10-23

**Authors:** Björn Berglund, Daphne Wezenberg, Maud Nilsson, Bo Söderquist, Lennart E. Nilsson, Jörg Schilcher

**Affiliations:** 1 Department of Biomedical and Clinical Sciences, Linköping University, Linköping, Sweden; 2 Department of Cell and Molecular Biology, Uppsala University, Uppsala, Sweden; 3 Department of Orthopedic Surgery, Linköping University Hospital, Linköping, Sweden; 4 Department of Laboratory Medicine, Clinical Microbiology, School of Medical Sciences, Faculty of Medicine and Health, Örebro University, Örebro, Sweden; 5 Wallenberg Centre for Molecular Medicine, Linköping University, Linköping, Sweden; NHLS Tygerberg/Stellenbosch University, Cape Town, Western Cape, South Africa

**Keywords:** antibiotics, bone graft, prosthetic joint infection, prophylaxis, tobramycin, vancomycin

## Abstract

Local delivery of antibiotics as prophylaxis for prosthetic joint infections (PJIs) is frequently used during total hip replacement surgery. Morselized bone allograft impregnated with vancomycin and tobramycin (*TobraVanc*) could provide effective prophylaxis against bacteria commonly associated with PJIs. In this study, the concentrations of antibiotics released by bone allograft impregnated with *TobraVanc* were determined by using an *in vitro* bioassay system entailing measuring inhibition zone diameters caused by antibiotic-impregnated bone chips cast in agar against standard curves. The concentrations were determined in samples of *TobraVanc*-impregnated bone graft taken before and after the application of the bone graft in the patients undergoing acetabular revision surgery. Antibiotic-impregnated bone grafts, sampled prior to application in the patient, delivered antibiotics in the concentration ranges of 730–9,800 mg/L for tobramycin and 1,300–11,000 mg/L for vancomycin. Samples taken after application in the patient released lower concentrations of tobramycin (490–1,900 mg/L; *P* < 0.01) and vancomycin (3,000–5,100 mg/L; *P* < 0.05); however, these concentrations remained well above the tobramycin minimum inhibitory concentrations (MICs) for investigated, highly tobramycin-resistant *Staphylococcus epidermidis* strains (MICs > 256 mg/L). At the tested concentrations, bone graft material mixed with *TobraVanc* delivered antibiotics in potent concentrations above the MICs for bacteria causing PJIs. Clinical trials are needed to evaluate the efficacy and risk of *TobraVanc*-impregnated bone graft as a prophylactic agent for patients undergoing hip replacement surgery.

**IMPORTANCE:**

Antibiotic prophylaxis is the cornerstone of successful joint replacement surgery, reducing the risk for the dreaded complication of prosthetic joint infection (PJI) to roughly 0.5%–2% in standard total hip replacement (THR). In addition to systemic antibiotics, antibiotics added locally have the potential to reduce the PJI risk even further, because of the high concentrations that can be achieved in the joint with limited risk for systemic toxicity. The results in the current study show that bone chips impregnated with a combination of tobramycin and vancomycin (*TobraVanc*) release antibiotics in concentrations that are potent against common bacteria causing PJIs. Especially in high-risk patients, our results support the prophylactic use of *TobraVanc* in hip replacement surgery requiring the use of a bone graft. A clinical study testing the efficacy of *TobraVanc*-impregnated bone graft in reducing the incidence of PJI in hip replacement surgery is currently ongoing (EudraCT: 2021-001708-14).

## INTRODUCTION

Total hip replacement (THR) to treat end-stage osteoarthritis is regarded as the most effective intervention to improve the quality of life for the affected individual (1–4). One of the most dreaded complications of THR is prosthetic joint infection (PJI). Approximately 0.5%–2.0% of joints become infected after primary hip replacement (5–7). To prevent PJIs, prophylactic antibiotics administered systemically have been used successfully for several decades (8, 9). In THR, the use of antibiotic-loaded bone cement generates high local concentrations of antibiotics in the joint, much higher than those achievable through intravenous administration (10). These high local concentrations may prevent the growth of microorganisms, as well as prevent the formation of microbial biofilms (6, 11), and have led to an additional 50% reduction in infection rates (5, 6, 12, 13). However, up to 95% of the antibiotic remains trapped inside the bone cement (14).

An alternative method to deliver high concentrations of antibiotics to the joint cavity during THR is to use a morselized bone graft as a carrier. Impacted morselized bone graft is used in primary and, more frequently, in revision THR surgeries to fill bone defects, so as to ensure stable fixation of the (revised) implant (15–19). Antibiotic-impregnated bone graft (AIBG) has superior elution kinetics compared to bone cement, yielding higher intra-articular concentrations than can be achieved with antibiotic-loaded bone cement (14, 20). Elevated serum levels of antibiotics after local application can result in systemic side effects, like nephrotoxicity and ototoxicity. A recent review has shown that in two-stage exchange surgery where antibiotic-loaded spacers are used, the rate of acute kidney injury might be higher than previously assumed (21).

The limited data available where the bone as the local carrier is investigated suggest that despite the very high antibiotic concentrations in the joint, the systemic uptake is negligible (11, 22, 23). Thorough monitoring of at-risk patients remains paramount independent of the carrier used. The AIBG might be of value not only for treating PJIs but also as adjuvant prophylaxis in patients who are at high risk of infection while undergoing joint revision surgery or complex primary joint replacement (24).

For use as prophylaxis, the antibiotic of choice to be mixed with the bone graft should be an agent that is effective against both gram-positive and gram-negative bacteria that are commonly associated with PJIs and without any negative systemic effects or interference with implant osseointegration (25). Aminoglycosides are examples of antibiotics that have been used in bone cement, which have activity against many bacteria causing PJIs (26). The aminoglycosides tobramycin and gentamicin are broad-spectrum antibiotics active against both gram-negative and gram-positive bacteria causing PJIs, with comparable antimicrobial spectra but with minor differences (27). Compared to gentamicin, tobramycin is more effective against *Acinetobacter baumannii* and *Pseudomonas aeruginosa*; however, these bacteria only rarely cause PJIs (28, 29). Vancomycin, on the other hand, is mainly used to treat infections caused by staphylococci, which are the most common bacteria causing PJIs (30).

Thus, a combination of tobramycin and vancomycin might be effective against most bacteria-causing PJIs (31–33), and the combination could have a synergistic effect on elution kinetics (34, 35). Moreover, vancomycin and tobramycin both show low osteotoxicity (36). In the current study, tobramycin and saline are used to dilute vancomycin in its ampule. This solution (*TobraVanc*) is then used during surgery to impregnate morselized bone grafts prior to impaction into bone defects.

The aims of the present study were to (i) determine the concentrations of antibiotics released by bone graft impregnated with *TobraVanc* by using an *in vitro* bioassay system and (ii) explore if the bone graft releases tobramycin in concentrations that are sufficiently high to be effective against highly aminoglycoside-resistant *Staphylococcus epidermidis*. We hypothesized that the bone graft impregnated with *TobraVanc* has a strong antimicrobial effect.

## MATERIALS AND METHODS

### Study setting and strain collection

In this study, a previously characterized collection of vancomycin-susceptible *S. epidermidis* (*n* = 139) from hip and knee PJIs was used (37). The isolates were collected between 2007 and 2016 in Region Örebro County and Region Östergötland, two administrative regions in central Sweden, each served by a single microbiology department. The isolates were preserved at −80°C, as per the clinical routine, in a preservation medium: trypticase soy broth (BD Diagnostic Systems, Hunt Valley, MD, USA), supplemented with 0.3% yeast extract (BD Diagnostic Systems) and 29% horse serum (Håtunalab AB, Bro, Sweden).

### Determination of aminoglycoside MICs with gradient tests and broth dilution

To determine which aminoglycoside would constitute the superior choice in the prophylactic mix between tobramycin and gentamicin in terms of MIC efficacy, MICs were determined for the *S. epidermidis* isolates. The isolates were first subcultured from storage at −80°C by inoculation on 3.8% (wt/vol) Mueller–Hinton agar (Oxoid, Basingstoke, Hampshire, England) and incubation at 35°C. MIC determinations were performed according to the European Committee on Antimicrobial Susceptibility Testing (EUCAST) guidelines (http://www.eucast.org) and were likewise interpreted according to the EUCAST-recommended break points (v 14.0). Gradient tests by using Etest (bioMérieux, Marcy l’Étoile, France) were used to determine MICs of tobramycin and gentamicin for all *S. epidermidis* isolates (*n* = 139). Isolates (*n* = 25) for which MICs of tobramycin were higher than the quantification range of the gradient test (MICs > 256 mg/L) were defined as highly resistant to tobramycin and were subjected to further MIC determinations by using a high-range broth microdilution assay. The isolates were suspended in NaCl (0.9% wt/vol) adjusted to 0.5 McFarland standard, serially inoculated in Mueller–Hinton broth in sterile glass tubes with a series of twofold dilutions of tobramycin (Thermo Fisher Scientific, Waltham, MA, USA) between 64 and 4,096 mg/L and incubated at 35°C. The tubes were visually inspected for bacterial growth after incubation for 24 h, and MICs were recorded. *Escherichia coli* ATCC 25922 was used as a control strain.

### Bone allograft as a carrier for *TobraVanc*

In the clinical routine at our institution, morselized bone allograft (25–55 g) is used as filler and scaffolding for defects in the acetabulum. Briefly, the bone graft is prepared from donated, fresh-frozen (−70°C), nonirradiated, allogenic femoral heads from the tissue bank, which were harvested from patients who underwent primary THR. The femoral heads were collected and stored according to the legal framework set out by the European Tissue and Cells Directive (Directive 2004/23/EC) and associated implementing Directives. During revision surgery, the donated femoral head is morselized in the operating theater using a Howex milling machine (Howex Corporation, Gävle, Sweden) to produce 3–5-mm bone chips. The bone graft is then repeatedly rinsed (minimum of two times) in saline, dried, and impregnated with *TobraVanc* and left to incubate for 10–30 minutes in the sterile environment of the operating theater. In this study, the *TobraVanc* solution was prepared by dissolving 1 g of vancomycin (Orion Pharma AB, Danderyd, Sweden) in 6 mL (480 mg) of tobramycin (generic version, Nebcina; Meda AB, Solna, Sweden) and 2 mL of saline. Mixing the bone graft with *TobraVanc* yields theoretical maximum concentrations of 60,000 mg/L for tobramycin and 125,000 mg/L for vancomycin. Samples of remnant bone graft chips impregnated with *TobraVanc* were collected from six femoral heads at two stages during acetabular revision surgery: (i) prior to application in the patient from the mixing bowl on the sterile side table (pre-application) and (ii) after application in the patients’ acetabular bone bed (post-application). The post-application bone chips have undergone impaction of the bone graft into the acetabular bed, rinsing of the wound with saline, trial evaluation, and impaction of the prosthesis. We chose to collect remnant samples at these two time-points because the surgical handling of the bone graft could potentially decrease the concentration of the antibiotics in the bone chips. To reduce between-patient variability, the pre-application and post-application samples were collected in pairs from the same patient during the same procedure (Fig. S1). The collected bone chips were placed in sterile plastic containers and stored at −70°C. All samples were thawed at room temperature prior to the experiments.

### Agar well bioassay for measuring tobramycin and vancomycin concentrations

To evaluate the concentrations of tobramycin and vancomycin released from the *TobraVanc*-impregnated bone chips, an agar well bioassay was established, modified from a previous study (38). For this, bacterial suspensions (0.5 McFarland) of either *E. coli* ATCC 25922 (intrinsically resistant to vancomycin) or the blood culture isolate *Enterococcus faecium* AB202561 (resistant to tobramycin; MIC > 4,096 mg/L) were spread on Mueller–Hinton agar plates. Thereafter, wells (~4 mm in diameter) were punched in the agar and filled with either 10 µL of antibiotic solution or 5–10 mg of chip samples from the AIBG. The inhibition zone diameters centered on the wells after incubation at 35°C for 24 h were measured, recorded, and related to antibiotic concentration released from the contents of the wells by a standard curve established as described below.

### Establishing standard curves to relate inhibition zone diameter to tobramycin and vancomycin concentrations

As a first step, data for establishing standard curves to relate the inhibition zone diameter to antibiotic concentration were obtained by using the agar well bioassay with wells that were filled with tobramycin (Nebcina; Meda) at concentrations of 60, 375, 600, 750, 1,500, 3,000, 6,000, and 60,000 mg/L and with vancomycin (Orion Pharma) at concentrations of 125, 780, 1,250, 1,560, 3,125, 6,250, 12,500, and 125,000 mg/L. The experiment was performed in duplicate with *E. coli* ATCC 25922 used for the tobramycin assay and *E. faecium* AB202561 used for the vancomycin assay.

We further determined whether the solution containing both tobramycin and vancomycin at high concentrations affected the inhibition zone diameters with the agar well bioassay compared to single antibiotic solutions. To do this, wells were filled with 10 µL of either tobramycin (Nebcina; Meda) at concentrations of 125, 1,250, 12,500, or 125,000 mg/L or vancomycin (Orion Pharma) at concentrations of 60, 600, 6,000, and 60,000 mg/L or a combination of both drugs at concentrations of 125 and 60, 1,250 and 600, 12,500 and 6,000, and 125,000 and 60,000 mg/L of tobramycin and vancomycin, respectively. The experiment was performed in triplicate, with all antibiotic concentrations, either singly or in combination, tested for both *E. coli* ATCC 25922 and *E. faecium* AB202561. NaCl (0.9% wt/vol) was used as a negative control.

### Concentrations of antibiotics delivered by bone chips impregnated with *TobraVanc*

To evaluate the concentrations of antibiotics delivered by bone chips impregnated with *TobraVanc*, bone chips (5–10 mg) from six femoral heads, sampled pre- and post-application of the bone graft in the patients, were cast in the wells of the agar well bioassay with melted agar added to the wells. After incubation at 35°C for 24 h, inhibition zone diameters for *E. coli* ATCC 25922 and *E. faecium* AB202561 were measured and converted to the corresponding tobramycin and vancomycin concentrations, respectively, by using the previously established standard curves. The experiment was performed in triplicate, and bone graft not exposed to antibiotics was used as a negative control. In addition, the milling process used to prepare the bone chips resulted in chips of different sizes and weights. To examine the influence of bone chip weight on antibiotic concentration, eight samples of antibiotic-impregnated bone chips of different weights (range, 5–10 mg) collected from a single femoral head were compared in the agar well bioassay.

### Statistical analysis

Differences between MIC distributions of tobramycin and gentamicin were determined with the Wilcoxon signed-rank test. Standard curves relating inhibition zone diameter to the logarithm of the antibiotic concentration were established by using linear regression. To determine whether the inhibition zone diameters (i.e., antibiotic concentration) of the AIBG differed between different donors and between sample types (pre- and post-application), a two-way ANOVA analysis was performed. To determine if the donated femoral head influenced the antibiotic concentration, a factor representing the different donors was added as a potential confounder in the analysis. The correlation between bone graft chip weight and inhibition zone diameter was determined by using Spearman’s rank correlation coefficients, where ρ < 0.5 was considered as not correlated. *P* < 0.05 was considered statistically significant for all tests. Statistical analyses were performed in GraphPad Prism ver. 9.1.2 (GraphPad Software Inc., San Diego, CA, USA).

## RESULTS

### Comparison of gentamicin and tobramycin MICs for *S. epidermidis*

**Fig 1:**
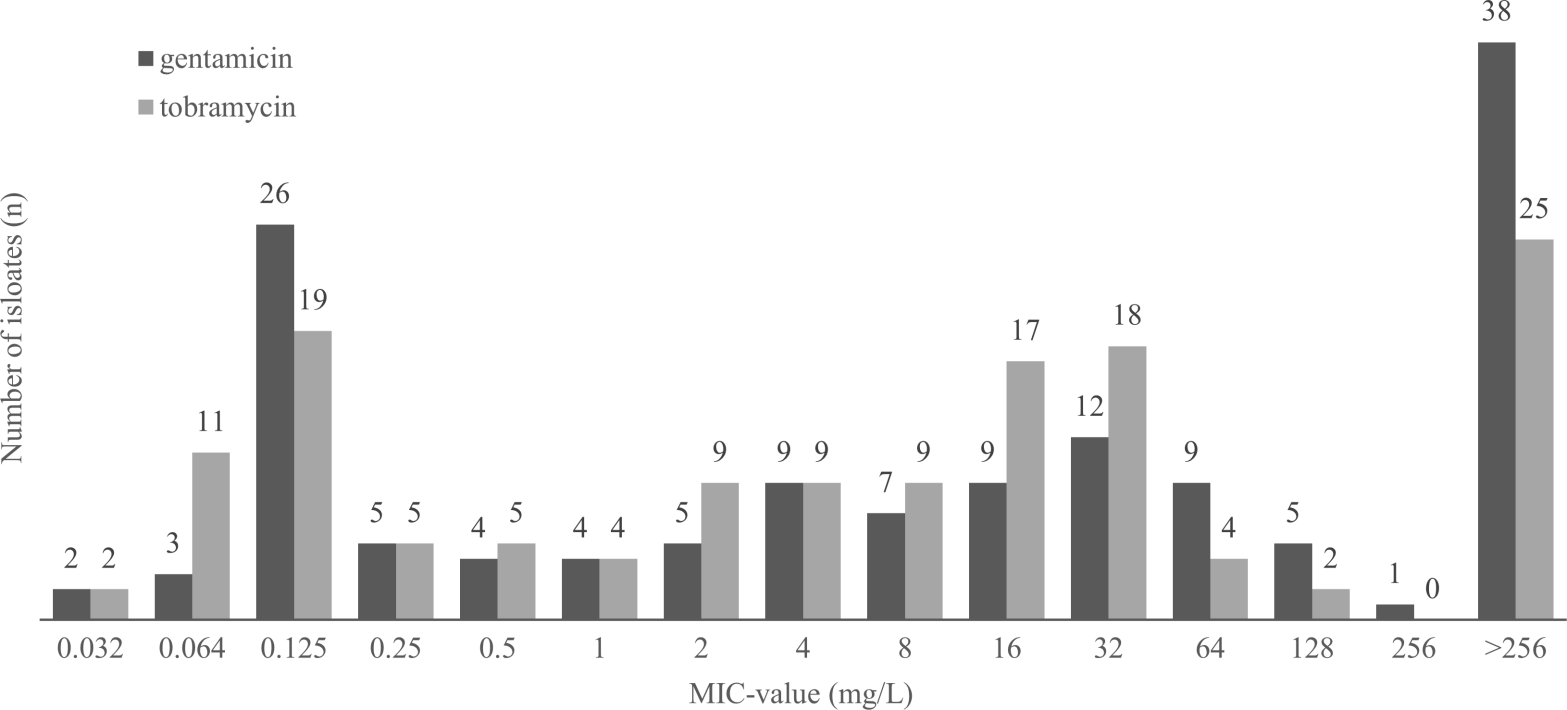
Distribution of the MICs of gentamicin and tobramycin, for 139 isolates of *S. epidermidis* obtained from patients with prosthetic joint infections in Sweden.

The distributions of MICs determined by the gradient test for gentamicin and tobramycin are shown in Fig. 1. Both aminoglycosides displayed MIC values that ranged from 0.032 to >256 mg/L, with tobramycin having significantly lower MICs (*P* < 0.001). The MIC_50_ and MIC_90_ values for gentamicin were 4 and 32 mg/L, respectively. The corresponding values for tobramycin were 4 and 16 mg/L, respectively. In total, 38 (27%) isolates were highly resistant (>256 mg/L) to gentamicin, as compared to 25 (18%) isolates that were highly resistant to tobramycin.

The tobramycin MICs for the 25 isolates that were highly resistant to tobramycin were further analyzed in a broth macro-dilution assay with a concentration range of 64–4,094 mg/L. Of these isolates, the MICs were ≤64 mg/L for 9 (36%), 128 mg/L for 10 (40%), and 256 mg/L for 6 (24%) isolates. MICs > 256 mg/L were not observed with the broth macro-dilution assay.

### Establishing standard curves and relating inhibition zone diameter to antibiotic concentration

**Fig 2:**
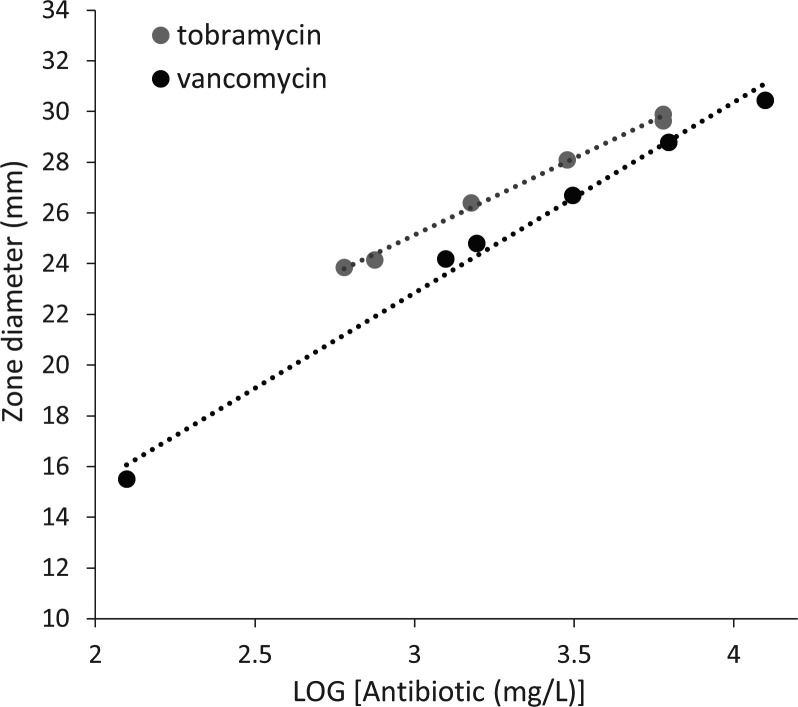
Standard curves relating the log concentrations of tobramycin and vancomycin to the inhibition zone diameter (mm). Each point on the dashed lines represents the mean of a duplicate observation. Linear regression lines are presented corresponding to the equation presented in the text.

Within the concentration intervals of 400–6,000 mg/L for tobramycin and 130–13,000 mg/L for vancomycin, the inhibition zone diameters were found to be linearly related to the logarithms of the concentrations of tobramycin (*R*^2^ = 0.97, *P* < 0.0001) and vancomycin (*R*^2^ = 0.99, *P* < 0.01) (Fig. 2) for the bioassay strains. The relationships between the log concentrations of tobramycin and vancomycin and the obtained zone diameters can be described as follows:


Zone diameter (mm)=6.3 ×log⁡[Tobramycin (mg/L)]+6.3,

Zone diameter (mm)=7.4 ×log⁡[Vancomycin (mg/L)]+0.6.

**Fig 3:**
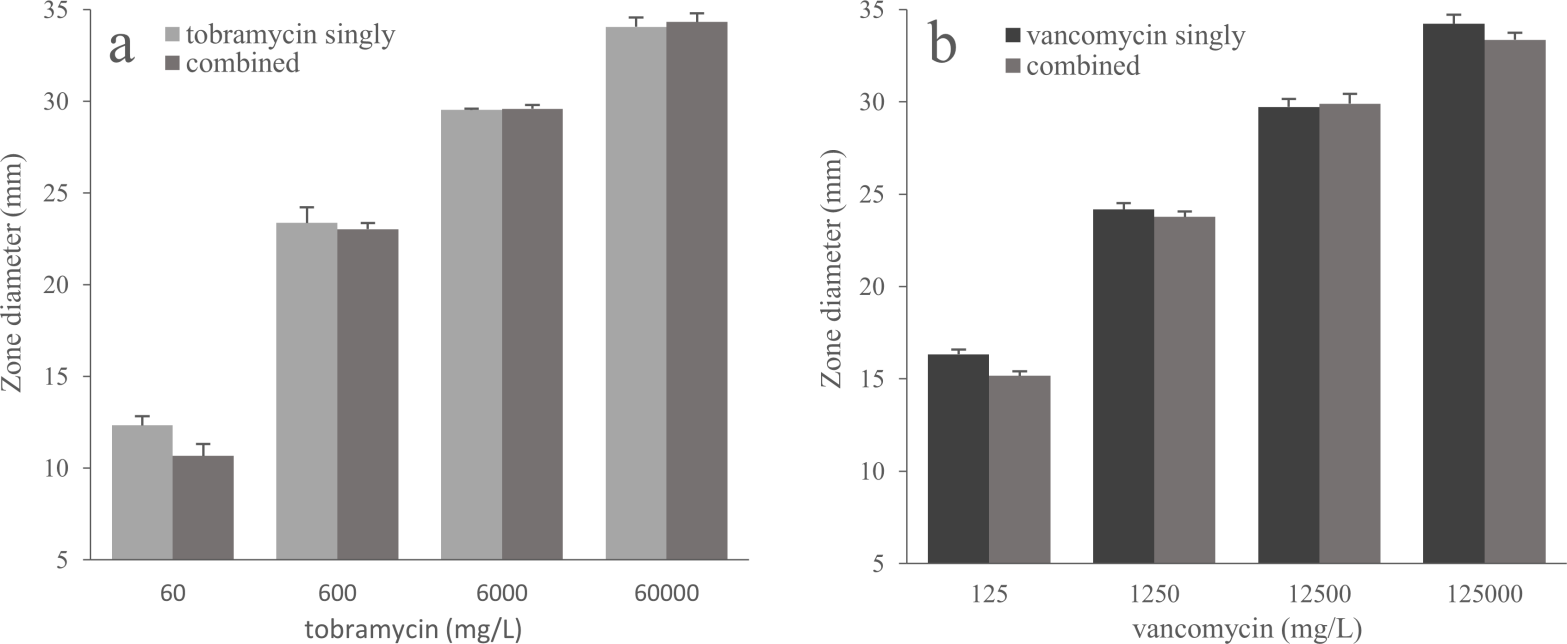
Antibiotic concentrations assessed by using inhibition zone diameter for tobramycin and vancomycin used singly, as compared to the combination of both antibiotics (combined) for a triplicate series of concentrations ranging up to 60,000 mg/L for tobramycin and 125,000 mg/L for vancomycin. The agar plates were inoculated with (a) *Escherichia coli ATCC 25922* (intrinsically resistant to vancomycin and susceptible to tobramycin) and (b) *Enterococcus faecium AB202561* (resistant to tobramycin and susceptible to vancomycin).

The inhibition zone diameters were on average 0.4 mm (SD = 1.4) and 0.6 mm (SD = 1.1) smaller for the combination of antibiotics than for either tobramycin alone or vancomycin alone (Fig. 3). The largest differences in inhibition zone diameter between the single and combined antibiotics were observed at the lower concentrations: the tobramycin concentration 60 mg/L (mean diameter = 1.7 mm; SD = 0.8) and the vancomycin concentration 125 mg/L (mean diameter = 1.2 mm; SD = 0.4). For comparison, the retest variability within measured triplicates was between 0.1 and 1.6 mm.

### Concentrations of antibiotics delivered by bone chips impregnated with *TobraVanc*

**Fig 4:**
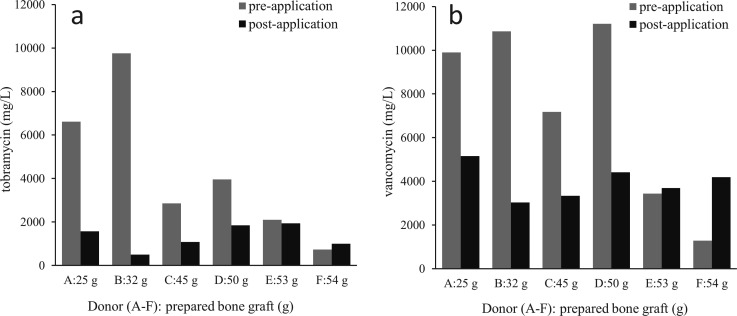
Differences in antibiotic concentrations for (a) tobramycin and (b) vancomycin between bone graft chips collected from the femoral heads of six different donors (donors A to F), with the total weights of the prepared bone graft chips for each donor on the *X*-axis. Data are presented from samples taken at two time-points during acetabular revision surgery: (i) prior to application in the patient (pre-application; light-gray bars) and (ii) after application in the acetabular bone bed (post-application; dark-gray bars).

For tobramycin, the measured inhibition zone diameters from the bone graft corresponded to concentrations in the range of 730–9,800 mg/L (mean = 3,200 mg/L) pre-application and 490–1,900 mg/L (mean = 1,200 mg/L) post-application. For vancomycin, this corresponded to a range of 1,300–11,000 mg/L (mean = 5,800 mg/L) pre-application and 3,000–5,100 mg/L (mean = 3,900 mg/L) post-application (Fig. 4). The inhibition zone diameters for the post-application bone grafts were significantly reduced compared to the pre-application bone grafts, for both tobramycin (*P* < 0.01) and vancomycin (*P* = 0.032).

In the weight interval of 5–10 mg, there was no significant correlation between the weight of the bone chip and the inhibition zone diameter (Fig. S1), irrespective of whether the agar plates were inoculated with the *E. coli* (*r* = 0.55, *P* = 0.16) or the *E. faecium* (*r* = −0.16, *P* = 0.72) strain. The variability associated with using femoral heads from different donors had a significant effect on the inhibition zone diameters for both tobramycin (*P* = 0.014) and vancomycin (*P* < 0.01) (Fig. S3).

## DISCUSSION

Our results support the hypothesis that bone allografts impregnated with *TobraVanc* deliver antibiotics in high concentrations. Our results show that the measured concentrations of tobramycin released from the *TobraVanc*-impregnated bone chips after application in the patient (1,200 mg/L post-application) were substantially higher than the highest MICs determined for the highly tobramycin-resistant strains of *S. epidermidis* collected from PJIs in Sweden (MIC = 256 mg/L). In the case of small colony variants (SCVs), which are prevalent in PJIs (39), it is not known what concentrations of antibiotics are needed for bacterial eradication. However, intraoperative contamination is the most important cause of infection (40), and the current results suggest that for prophylactic purposes, local delivery by using *TobraVanc-*impregnated bone grafts, with the goal of achieving high local concentrations of the antibiotics, might reduce the risk of PJIs and the subsequent development of SCVs and biofilms.

The concentrations of antibiotics delivered by antibiotic-impregnated bone chips post-application in the patient (sampled after the bone bed was prepared and rinsed) were lower for both tobramycin and vancomycin, as compared to the samples taken pre-application in the patient (~2.5-fold and 1.5-fold lower concentrations, respectively). Cleaning of the surgical area, including the bone graft bed, with saline by pulsed lavage likely influenced the antibiotic concentrations in the remnant bone chips collected post-application, especially since the bone graft remnants were from the superficial layers of the bone graft bed, which is more exposed to the lavage fluid. However, the mean concentrations of antibiotics released from chips collected post-application (1,200 mg/L for tobramycin and 3,900 mg/L for vancomycin) were still considerably higher than the MICs for bacteria-causing PJIs. Therefore, lavage of the wound with saline is not likely to adversely affect the prophylactic efficacy of the antibiotic-impregnated bone chips, and it can be performed simultaneously as a cost-effective method to further reduce the risk of PJIs after THR surgery (41).

Tobramycin and gentamicin are aminoglycosides that are frequently used for local antibiotic treatment. Together, *Staphylococcus aureus* and *S. epidermidis* comprised about 60%–80% of all PJIs in Sweden (32), and while *S. aureus* is rarely resistant to aminoglycosides, *S. epidermidis* are frequently highly resistant to these antibiotics (42). To substantiate the choice of aminoglycoside for use in the prophylactic mixture to be blended with bone grafts, we compared the MICs of tobramycin and gentamicin for 139 clinical isolates of *S. epidermidis* collected from patients with PJIs in Sweden for a previous study (37). In terms of MIC distribution, tobramycin was superior to gentamicin and was thus selected as the aminoglycoside to be used together with vancomycin as the local prophylactic antibiotic mix in bone grafts. According to whole-genome sequencing data presented for the strain collection in a previous study (37), the *S. epidermidis* obtained from PJIs were found to be oligoclonal, with 43% belonging to sequence type (ST) 2 and 25% belonging to ST215. Out of the 85 *S*. *epidermidis* isolates that displayed MICs ≥ 4 mg/L for tobramycin, only 6 did not belong to either ST2 or ST215. The ST2 lineage comprised only isolates with MICs > 256 mg/L, according to the gradient test. The *aacA-aphD* and *aadD* genes, which encode resistance to aminoglycosides, were found in 64% (16/25) of the isolates displaying an MIC >256 mg/L to tobramycin, as compared to 24% (27/114) of the isolates with MICs ≤ 256 mg/L. Isolates that carried only one gene encoding aminoglycoside resistance were represented by *aacA-aphD* in most cases. Consequently, *S. epidermidis* strains displaying highly resistant to aminoglycosides correspond to specific lineages (STs) with multiple aminoglycoside resistance genes. The antibiotic concentrations achieved with the *TobraVanc-*impregnated bone graft are well above these MICs, demonstrating the potential to be effective even against the most resistant strains of *S. epidermidis* in PJIs. However, the 139 clinical isolates of *S. epidermidis* were obtained from patients with PJIs in a limited geographic area of Sweden. For these isolates, tobramycin outperformed gentamicin. However, because of the phenotypic and genotypic diversity of PJI isolates worldwide, the generalizability of the results might be limited.

Using a dual-antibiotic treatment (tobramycin and vancomycin) ensures effectiveness against most of the bacteria that cause PJIs (31–33). Previous studies using acrylic bone cement have shown a synergistic effect of combining tobramycin and vancomycin, which is most likely a result of the improved elution kinetics of vancomycin from bone cement (34, 35). Further research is required to determine whether or not similar synergistic effects might be present when bone graft is used as a carrier. The local application of antibiotics and their effects on tissue regeneration have been studied extensively. At high concentrations, some antibiotics can have negative effects on bone healing related to their toxic effects on osteoblasts and other bone cells involved in bone remodeling during osseointegration (24). Even other cell types of the connective and muscular tissues are negatively affected in dose- and time-dependent manners (43). However, several studies have investigated antibiotic-related osteotoxicity, and the consensus is that vancomycin and tobramycin are among the antibiotics that show the lowest cytotoxicity levels for osteoblasts, pre-osteoblasts, and pre-chondrocyte cell lines. Even if there appear to be some negative effects of locally applied antibiotics *in vitro*, there are no verified negative effects of tobramycin or vancomycin on bone graft incorporation or implant osseointegration (44–47).

The current study shows that combining tobramycin and vancomycin does not negatively influence the concentrations of antibiotics released by either antibiotic. In bone cement, the release of antibiotics is mainly from the surface, and a large proportion of the antibiotic remains trapped inside the cement, thereby creating the risk of low antibiotic concentrations in the joint fluid, which might lead to the development of bacterial resistance. A more burst-like release of antibiotics from the bone graft during the first 24 h, followed by gradual release into the joint over the course of up to 14 days (48), might be more effective than the low-grade release from bone cement (20). How much antibiotic is released does not seem to be related to the weight of the sampled bone graft chips within the weight interval tested (5–10 mg). It is possible that the total surface area available for the adhesion of antibiotics has a more important influence on the elution kinetics (49). Longitudinal sampling of antibiotic concentration was not performed. Therefore, long-term elution kinetics of *TobraVanc-*impregnated bone graft was not investigated, and the potential presence of subinhibitory concentrations of antibiotics in the joint and the consequent risk of bacterial resistance induction remain unknown (20).

This study investigated bone grafts impregnated with tobramycin and vancomycin prior to application in the patient and after application in the patient to understand the effect of the surgical handling on the concentrations of antibiotics released from the graft. As expected, the antibiotic concentrations decreased after the surgical handling of the bone graft in the patient, although the dosage still yielded concentrations above the MICs of the highly tobramycin-resistant strains. Based on these results, bone grafts impregnated with *TobraVanc* at the antibiotic concentrations used in these experiments are likely to release antibiotics locally at concentrations potent against bacteria that cause PJIs. In addition, the high local concentrations of antibiotics are expected to affect even highly resistant strains. While high, local concentrations are ideal to eradicate bacteria, there is no concluding evidence available on the potential risks for adverse events related to systemic toxicity. A multicenter randomized clinical trial (EU Clinical Trial Register: 2024-510921-25-00) is ongoing, which aims to evaluate the efficacy and risks of *TobraVanc*-impregnated bone graft as a prophylaxis to reduce PJIs in patients after THR surgery.

## Data Availability

Data that support the findings of this study are available from the corresponding author upon reasonable request.

## SUPPLEMENTAL MATERIAL

Figure S1 spectrum.00414-24-s0001.png Flowchart describing the preparation and impregnation of the bone graft.

Figure S2 spectrum.00414-24-s0002.png Inhibition zone diameters measured in agar.

Figure S3 spectrum.00414-24-s0003.png Inhibition zone diameters measured in agar inoculated with the total weights of the prepared bone graft chips for each donor.

Supplemental legends spectrum.00414-24-s0004.docx Legends for Fig. S1 to S3.

ASM does not own the copyrights to Supplemental Material that may be linked to, or accessed through, an article. The authors have granted ASM a non-exclusive, world-wide license to publish the Supplemental Material files. Please contact the corresponding author directly for reuse.
