# Supplementary figures and images for: Bone allograft impregnated with tobramycin and vancomycin delivers antibiotics in high concentrations for prophylaxis against bacteria commonly associated with prosthetic joint infections
Source: Microbiol Spectr. 2024 Oct 23;12(12):e00414-24. doi: 10.1128/spectrum.00414-24 (PMC11619462; doi:10.1128/spectrum.00414-24)

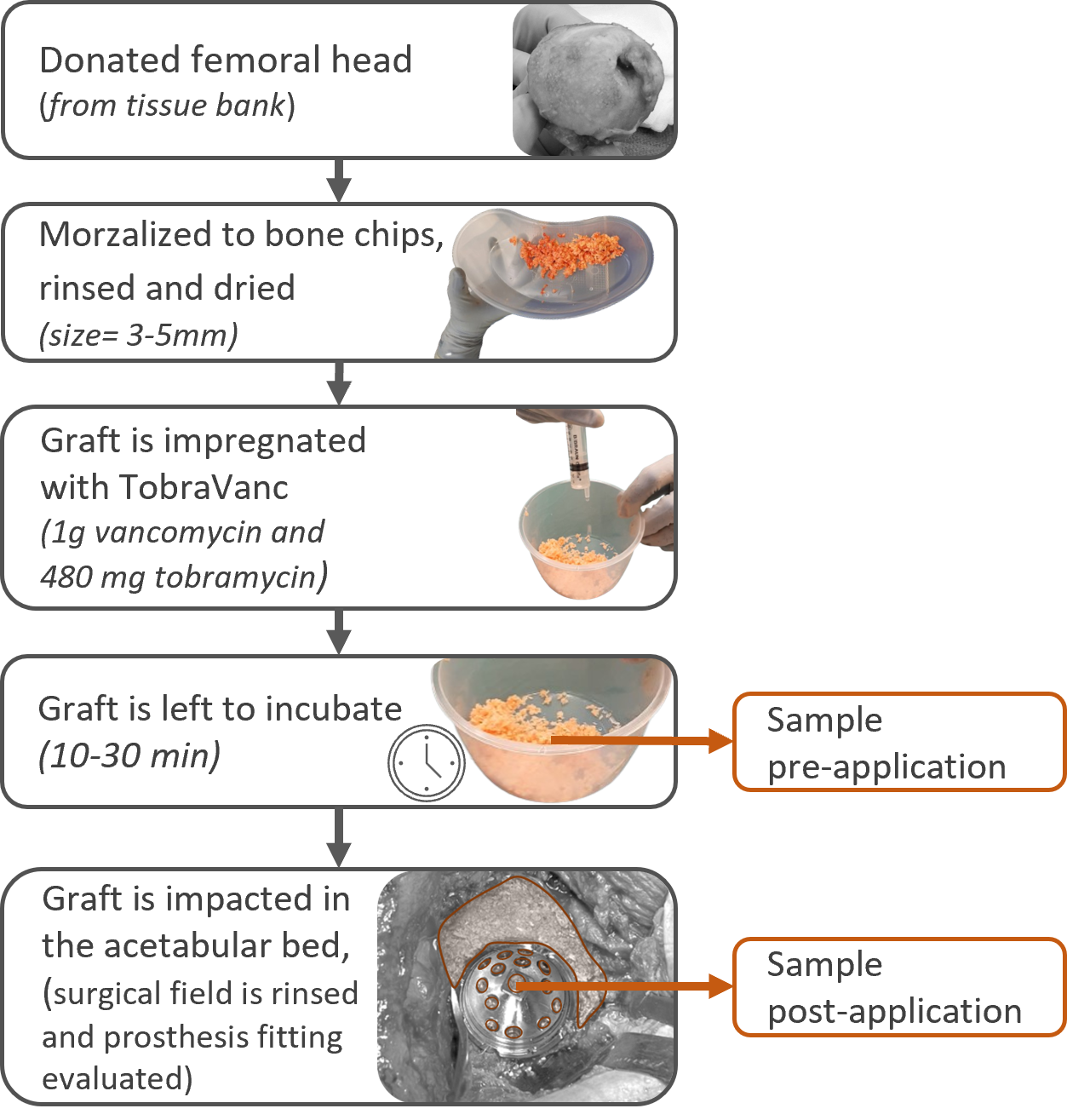

Supplement: Figure S1 — Flowchart describing the preparation and impregnation of the bone graft. [file spectrum.00414-24-s0001.png]

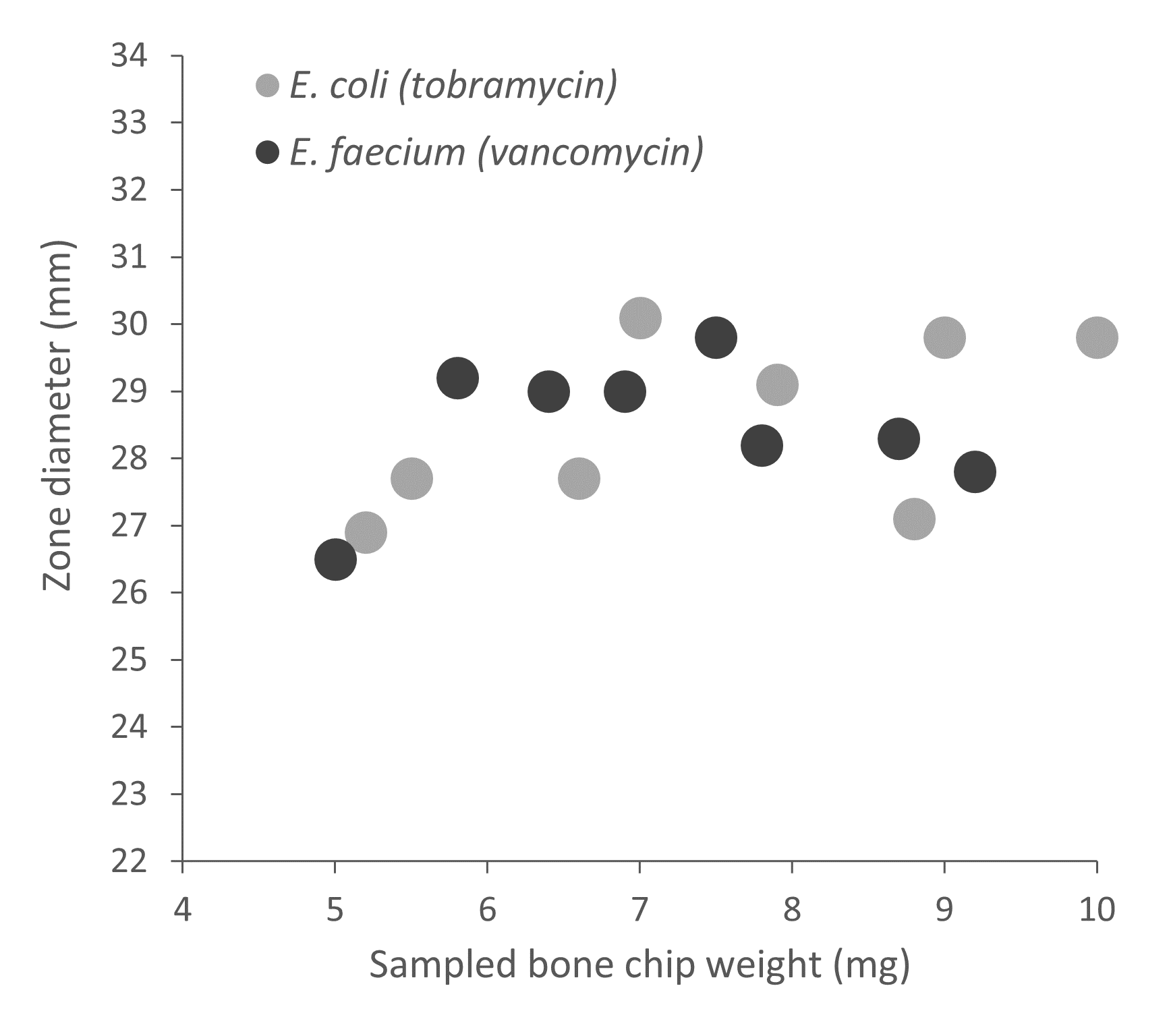

Supplement: Figure S2 — Inhibition zone diameters measured in agar. [file spectrum.00414-24-s0002.png]

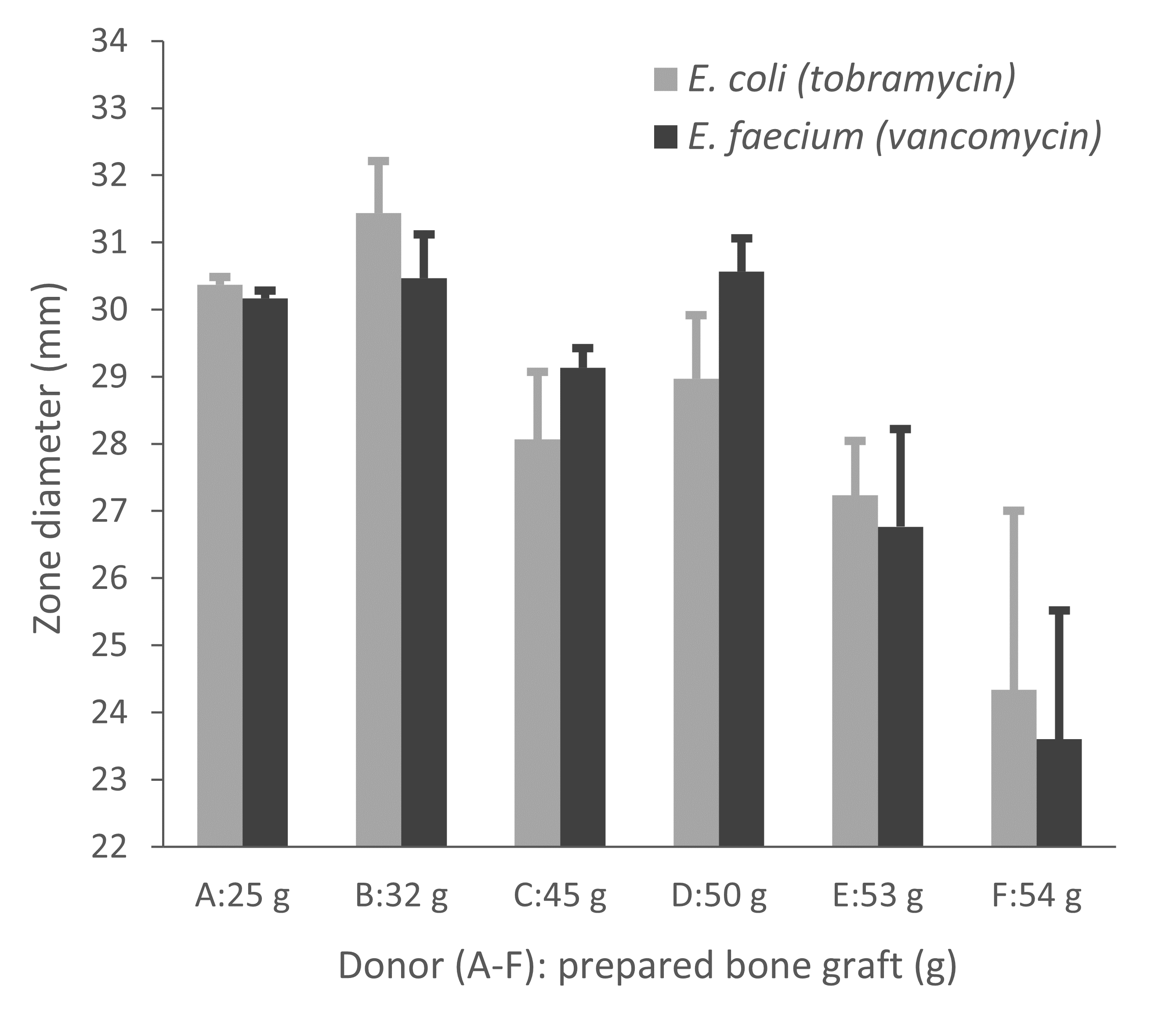

Supplement: Figure S3 — Inhibition zone diameters measured in agar inoculated with the total weights of the prepared bone graft chips for each donor. [file spectrum.00414-24-s0003.png]
