# Supplementary material for: Bone allograft impregnated with tobramycin and vancomycin delivers antibiotics in high concentrations for prophylaxis against bacteria commonly associated with prosthetic joint infections
Source: Microbiol Spectr. 2024 Oct 23;12(12):e00414-24. doi: 10.1128/spectrum.00414-24 (PMC11619462; doi:10.1128/spectrum.00414-24)
Supplement: Supplemental legends — Legends for Fig. S1 to S3. [file spectrum.00414-24-s0004.docx]

**Supplement Figure Headings**

**FIG S1** Flowchart describing the preparation and impregnation of the bone graft with tobramycin and vancomycin in the operating theatre during total hip replacement surgery. Samples of remnant bone graft chips were collected from the preparation bowl (pre-application) and from bone graft that was superfluous in the screw holes after application of the acetabulum cup in the patient (post-application). The area where bone graft is applied is indicated in orange.

**FIG S2** Inhibition zone diameters measured in agar inoculated with *Escherichia coli* ATCC 25922 (intrinsically resistant to vancomycin and susceptible to tobramycin; gray dots/bars) or *Enterococcus faecium* AB202561 (resistant to tobramycin (MIC>4,096 mg/L) and susceptible to vancomycin; black/bars dots) and *TobraVanc*-impregnated bone chips of different weights (mg) originating from one femoral head donor.

**FIG S3** Inhibition zone diameters measured in agar inoculated with *Escherichia coli* ATCC 25922 (intrinsically resistant to vancomycin and susceptible to tobramycin; gray bars) or *Enterococcus faecium* AB202561 (resistant to tobramycin (MIC>4,096 mg/L) and susceptible to vancomycin; black bars) and *TobraVanc*-impregnated bone chips from femoral heads from six different donors (A-F) with the total weights of the prepared bone graft chips for each donor on the X-axis (g).
